# Analysis of the EMHD nanofluid flow for geothermal pipelines using physics-driven deep learning

**DOI:** 10.1038/s41598-025-23315-1

**Published:** 2025-11-11

**Authors:** Faiza, Waseem, Saeed Islam, Flah Aymen, Mutum Zico Meetei, M. Mohamed

**Affiliations:** 1 Abdul Wali Khan University Mardan, Mardan, 23200 Pakistan; 2 School of Mechanical Engineering, Jiangsu University, Zhenjiang, 212013 Jiangsu P.R. China; 3 Department of Mechanical Engineering, Prince Mohammad Bin Fahd University, P.O Box 1664, Al Khobar, 31952 Kingdom of Saudi Arabia; 4 Centre for Research Impact and Outcome, Chitkara University Institute of Engineering and Technology, Rajpura, 140401 Punjab India; 5 Applied Science Research Center, Applied Science Private University, Amman, 11931 Jordan; 6 ENET Centre, CEET, VSB-Technical University of Ostrava, Ostrava, Czech Republic; 7 Department of Mathematics, College of Science, Jazan University, P.O. Box 114, 45142 Jazan, Saudi Arabia; 8 Mathematics Education Program, Faculty of Education and Arts, Sohar University, Sohar 311, Oman

**Keywords:** Electro-magneto-hydrodynamics (EMHD), Physics-informed neural networks (PINNs), Heat transfer enhancement, Hybrid intelligent modeling, Computational fluid dynamics (CFD), Engineering, Mathematics and computing, Physics

## Abstract

In recent years data-driven machine learning techniques attract the attention of researchers in analyzing many complex systems. This study introduces a novel unsupervised deep neural network approach to predict the temperature and velocity behaviour of electro-magneto-hydrodynamics hybrid nanofluid flow for geothermal pipelines application.The exceptional flow and thermal characteristics of hybrid nanofluids making them ideal for use in geothermal energy extraction applications. The dynamics of hybrid nanofluid flow through a pipe are examined using a third-grade sodium alginate model, which has a lot of potential for geothermal applications. The copper oxide (CuO) and zinc oxide (ZnO) nanoparticles make up the nanofluid. It is also investigated how the flow dynamics are affected by electric and magnetic fields. The energy equation takes into account the effects of Joule heating and viscous dissipation as the fully developed incompressible fluid passes through the pipe. Consequently, an unsupervised deep neural network (DNN) method is used to predict the dynamics of nonlinear differential equations (DEs). The accuracy of the deep neural network ranges from $10^{-06}$ to $10^{-13}$ across different cases. The velocity profile exhibits a clear symmetrical pattern and is found to be significantly influenced by both the electric field and the thermal Grashof number. The overall thermal profile along the pipe’s length decreases as a result of the nanoparticles. Additionally, lowering the pressure has a similar effect on both velocities. This study makes important contributions to the comprehension of the intricate dynamics of electro-magneto-hydrodynamics hybrid nanofluid flow, thereby laying a foundational framework for optimizing thermodynamic systems in geothermal. The findings of this research hold significant practical implications for the design and engineering of systems aimed at energy conservation and improved heat transfer efficiency in geothermal pipelines.

## Introduction

Over 28 years ago, nanofluids, a new category of fluids, were introduced by Choi and Eastman^1^, who enhanced thermal conductivity using water and copper nanofluids. Nanofluids^2–4^ are formed by suspending nanoparticles, which are particles less than 100nm, into traditional base fluids. This suspension of nanoparticles is produced with the primary intention of increasing the heat transfer capability of the base fluid. Historically, an array of base fluids has been tried like oils, waters, ethylene glycol and many other organic compounds and even lubricants. At the same time, a wide range of metal and carbon containing nanoparticles, including metal nitrides, metal oxides, and graphite have been incorporated.

Due to the benefits of nanofluids over conventional fluids, they have been integrated into various thermal technologies such as radiators, heat exchangers, solar energy systems, microelectronics cooling, and in some industries like oil, gas and automotive. The most advanced type of nanofluids, hybrid nanofluids, is a recent development in nanotechnology. These nanofluids consist of at least two different types of nanoparticles, each with an average size of less than 100 nanometers (nm), suspended in a base fluid. They possess superior thermal and physical properties. Hybrid nanofluids^5,6^ are characterized by their superior performance, enhanced thermal networks, synergies, and materia.

Important hybrid nanofluids can be tailored for various technical applications, notably in geothermal energy systems^7–9^. As an example, hybrid nanofluids can serve as fracture fluids in enhanced geothermal systems, where they improve the thermal and hydraulic conductivity of the fractures. Geothermal power plants require effective cooling systems to control the excess thermal energy produced. The efficiency of the cooling process is enhanced by using hybrid nanofluids as coolants. Their enhanced heat transfer characteristics yield heat dissipation benefits, greater efficiency in power generation, and a lesser negative impact on the environment. Moreover, hybrid nanofluids can also serve as drilling fluids for geothermal drilling^10^. The incorporation of nanoparticles into drilling fluids has demonstrated an augmentation of the lubrication properties, aiding in friction and tool wear mitigation.

Using hybrid nanofluids in geothermal systems enhances heat transfer efficiency tremendously owing to their remarkably high thermal conductivity^11^. Geothermal energy extraction becomes more feasible with these hybrid nanofluids, as they can easily circulate through geothermal wells or heat exchangers. Furthermore, it is necessary to address corrosion mitigation in geothermal systems because the fluids typically contain corrosive materials that threaten the integrity of pipelines and machinery^12^.

The analysis of numerical solutions for velocity and temperature distributions was conducted through a computational approach. Korei et al.^13^ investigated mixed convection and entropy generation in the context of magnetohydrodynamics (MHD) within a hybrid nanofluid flow comprising copper and aluminum oxide nanoparticles. Their study focused on a partially heated, chamfered lid-driven cavity. They demonstrated that application of a magnetic field enhances heat transfer, and the mixture of copper oxide ($25\%$) and aluminum oxide ($75\%$) significantly increases the Nusselt number and entropy generation. Kumbhakar and Nandi^14^ conducted a comprehensive study on the thermal radiative flow of magnetized hybrid nanofluids, utilizing copper and aluminum oxide nanoparticles. Their findings indicated that both slip velocity and Hartree pressure differentials lead to a decrease in fluid flow, while simultaneously elevating the temperature profile due to an increase in the Biot number and thermal radiation effects. In a separate investigation, Xia et al.^15^ analyzed the three-dimensional flow of hybrid nanofluids over an elastic surface, incorporating a variety of microorganisms. Their research also examined the effects of activation energy, joule heating, and slip BCs. Acharya^16^ investigated that movement of magnetized hybrid nanofluids through cylindrical apparatus equipped with fins. They employed the finite element method to produce numerical solutions for silver nanoparticles and magnesium oxide. In a separate study, Asghar et al.^17^ examined the flow characteristics of hybrid nanofluids containing copper and aluminum oxide nanoparticles, focusing on heat absorption, mixed convection and generation, slip conditions, and the dynamics of MHD. As stated by Animassam et al.^18^, hybrid nanofluids possess the potential to transform various industries through their superior thermal and material characteristics, resulting in improved energy efficiency, enhanced medical treatments, and groundbreaking technological innovations. Recent research concerning the magnetic effects of hybrid nanofluids can be found in^19–23^ references.

Shahmir et al. study the impact of externally applied magnetic fields on the movement of ferromagnetic ternary and hybrid nanofluids with surface catalyzed reaction and an assessment of entropy generation and a comparative evaluation of different thermal conductivity and viscosity models in hybrid nanofluid flow-a non-similar solution^24,25^. Kumar et al. presents the prediction of heat transfer in water-based hybrid nanofluid thin film flow and its optimization using Levenberg-Marquardt algorithm and Deep Learning methods, along with the forecasting of heat and mass transfer enhancement in magnetized non-Newtonian nanofluids^26,27^. The work of M Bilal et al. highlights the pioneering efforts of riga plate with novel numerical and artificial neural computing^28,29^.

Physics-informed machine learning is a developing area that is expected to significantly impact science and engineering in the long run. The PINNs^30,31^ shows outstanding efficiency with a diverse set of problems governed by PDEs^32,33^. To approach the solution of a differential equation, PINNs make use of the known universal approximation capabilities of neural networks^33^. Unlike standard numerical methods, PINNs construct a solution across an entire irregular domain. Commonly used deep learning frameworks universally implement standard gradient descent algorithms, ADAM^34^, as well as stochastic gradient techniques^35^ for training deep neural networks. These algorithms are also implemented when training Physics-Informed Neural Networks (PINNs).More recently, studies have pointed out that the gradient descent method does not follow a typical trend while training Physics-Informed Neural Networks (PINNs), especially when dealing with irregularities in the dataset for the associated differential equation^36^. To mitigate this issue, some remedies have been suggested, including the loss of weight technique^37^ and domain decomposition^38^.

The primary objective of this research is to explore the mechanisms of hybrid nanofluids composed of a sodium alginate base fluid combined with zinc oxide and copper oxide nanoparticles (ZnO/CuO-SA). The central focus and innovation of this study lie in examining the electro-magneto-hydrodynamic effects on hybrid nanofluids, utilising physics-driven deep learning method, a topic that has not been previously investigated in the context of geothermal energy extraction. The present simulation conducts a comprehensive analysis of the flow characteristics of non-Newtonian third-grade sodium alginate fluid incorporating hybrid nanoparticles. It investigates the effects of electric and magnetic fields through the application of energy and momentum equations.

## Methodology and problem setup

**Fig. 1:**
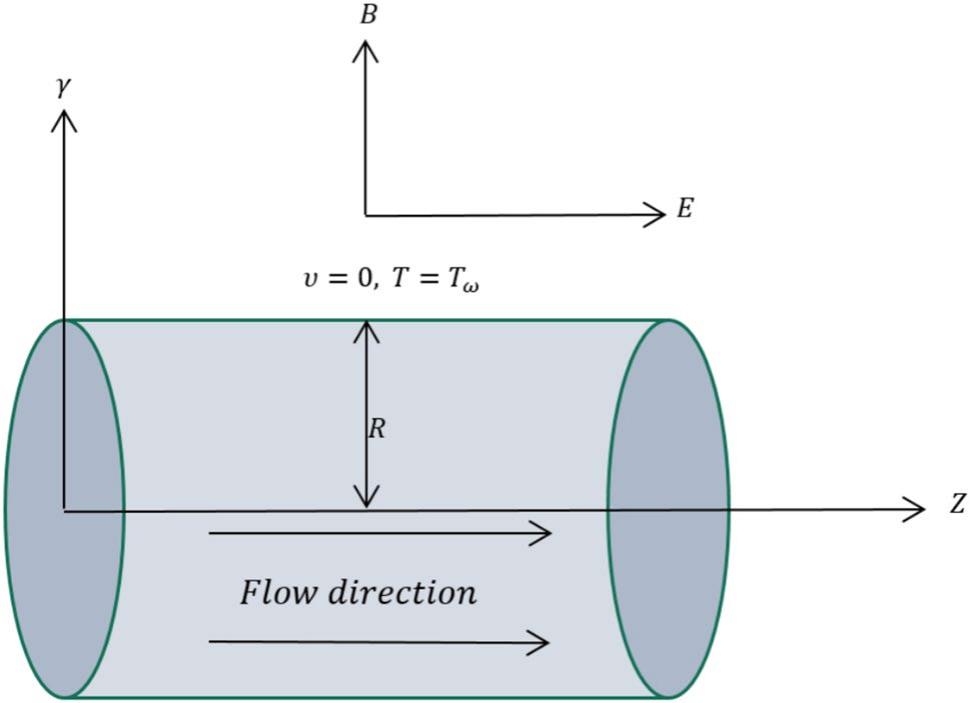
Geometry of the given problem.

Consider a fully developed incompressible flow of a hybrid nanofluid within a pipe containing zinc oxide and copper oxide nanoparticles (ZnO-CuO/ sodium alginate. The base fluid, identified as sodium alginate (SA), is classified as a third-grade fluid. The nanofluid is regarded as electrically conductive due to the influence of an applied electric field and a transverse magnetic field. Additionally, it is assumed that the nanoparticles and the base fluid maintain thermal equilibrium, resulting in the absence of slip. For the geometric configuration illustrated in Fig. 1, cylindrical coordinates are utilized, represented as $(z,0,z)$, where the $z$-axis is aligned with the centerline of the pipe and $r$ denotes the radial direction. The proposed mathematical model, which includes the continuity equation, momentum equation, and energy equation in cylindrical coordinates, is articulated as follows:

1 $$\begin{aligned} & \nabla \cdot \vec {V} = 0, \end{aligned}$$

2 $$\begin{aligned} & \rho _{\text {hnf}} \frac{d\vec {V}}{dt} = \text {div}\vec {\tau } + \vec {J} \times \vec {B} + g(\rho \beta )_{\text {hnf}}(T - T_w), \end{aligned}$$

In the equation presented above, $V$ denotes the velocity field vector, with the subscript $\text {inf}$ indicating hybrid nanofluid. The symbol $\rho$ signifies the density, while $d/dt$ represents the material time derivative. The variable $B$ stands for the magnetic field, $g$ refers to gravity, and $\beta$ indicates thermal expansion. $T$ represents temperature, $T_w$ is the temperature of the pipe, and $J = [\sigma _{hnf}(E + V \times B)]$ denotes the current density. Here, $\sigma$ is the electrical conductivity, $E$ is the electric field, and $\tau$ is the stress tensor associated with a third-grade fluid, which can be expressed as follows:

3 $$\begin{aligned} \vec {\tau } = -pI + \mu _{\text {hnf}}Z_1 + \xi _1Z_2 + \xi _2Z_1^2 + \eta _1Z_3 + \eta _2(Z_1Z_2 + Z_2Z_1) + \eta _3(\text {tr}Z_1^2)Z_1, \end{aligned}$$

In this context, $\mu$ denotes the viscosity, while $\xi _1,\xi _2,\eta _1,\eta _2,\eta _3$ represent the material moduli, which are typically dependent on temperature. Additionally, $pI$ signifies the spherical stress resulting from incompressibility constraints, along with the kinematical tensors, which are defined as follows.

4 $$\begin{aligned} \left. \begin{aligned}&Z_1 = (\text {grad}\vec {V}) + (\text {grad}\vec {V})^T \\&Z_i = \frac{d}{dt}Z_{n-1} + Z_{i-1}(\text {grad}\vec {V}) + (\text {grad}\vec {V})^T Z_{i-1}, i = 2,3,... \end{aligned}\right\} , \end{aligned}$$

The literature indicates that^24^, when all-fluid motion aligns with thermodynamic principles by satisfying the Clausius-Duhem inequality, and assuming that the specific Helmholtz free energy reaches its minimum when the fluid is locally at rest, the subsequent conditions are established as follows:

5 $$\begin{aligned} \mu \ge 0, \xi _1 \ge 0, |\xi _1 + \xi _2| \le \sqrt{24\eta _3\mu }, \eta _1 = \eta _2 = 0, \eta _3 \ge 0. \end{aligned}$$

For the current analysis, it is assumed that the fluid is thermodynamically compatible; consequently, Eq. (3) is expressed in the following manner.

6 $$\begin{aligned} \vec {\tau } = -pI + \mu _{\text {hnf}}Z_1 + \xi _1Z_2 + \xi _2Z_1^2 + \eta _3(\text {tr}Z_1^2)Z_1. \end{aligned}$$

It is important to note that the constitutive relation indicates a shear-thickening mechanism ($\eta _3 > 0$), which results in an increase in viscosity as the shear rate and normal stress differences rise. The energy equation, which incorporates the effects of joule heating and viscous dissipation, is expressed as follows:

7 $$\begin{aligned} \rho _{\text {hnf}} \frac{d\varepsilon }{dt} = \vec {\tau } \cdot \textbf{L} + \frac{\vec {J} \cdot \vec {J}}{\sigma _{\text {hnf}}} - \text {div} \textbf{Q}, \end{aligned}$$

where $\varepsilon$ denotes the specific internal energy, $L$ signifies the gradient velocity, and $Q$ refers to the heat flux vector, which can be articulated through Fourier’s law.

8 $$\begin{aligned} \text {div} \textbf{Q} = -\kappa _{\text {hnf}} \text { grad } T, \end{aligned}$$

where $\kappa _{\text {hnf}}$ represents the thermal conductivity. The existing formulation presumes that the velocity distribution and temperature profile are represented in the subsequent manner.

9 $$\begin{aligned} \vec {V} = v(\vec {r})e_{\vec {z}} \quad T = T(\vec {r}). \end{aligned}$$

Considering Eq. (9), the equations for momentum and energy are identified as follows:

10 $$\begin{aligned} & \frac{1}{\vec {r}} \frac{d}{d\vec {r}} \left[ \vec {r}(2\xi _1 + \xi _2) \left( \frac{dv}{d\vec {r}} \right) ^2 \right] = \frac{\partial p}{\partial \vec {r}}, \end{aligned}$$

11 $$\begin{aligned} & 0 = \frac{\partial p}{\partial \vec {\theta }}, \end{aligned}$$

12 $$\begin{aligned} & \frac{\mu _{\text {hnf}}}{\vec {r}} \frac{d}{dr} \left( \vec {r} \frac{dv}{d\vec {r}} \right) + \frac{1}{\vec {r}} \frac{d}{d\vec {r}} \left[ 2\beta _3 \vec {r} \left( \frac{dv}{d\vec {r}} \right) ^3 \right] - \sigma _{\text {hnf}} B_0^2 v + \sigma _{\text {hnf}} E B_0 + (\rho \beta )_{\text {hnf}}(T - T_w) = \frac{\partial p}{\partial z}, \end{aligned}$$

13 $$\begin{aligned} & \frac{\kappa _{\text {hnf}}}{\vec {r}} \frac{d}{d\vec {r}} \left( \vec {r} \frac{dT}{d\vec {r}} \right) + \mu _{\text {hnf}} \left( \frac{dv}{d\vec {r}} \right) ^2 + 2\beta _3 \left( \frac{dv}{d\vec {r}} \right) ^4 + \sigma _{\text {hnf}} B_0^2 v + \sigma _{\text {hnf}} E^2 - 2\sigma _{\text {hnf}} E B_0 v=0, \end{aligned}$$

The boundary conditions are defined as:

14 $$\begin{aligned} v = 0, T = T_w, \quad \text {at} \quad \vec {r} = R, \end{aligned}$$

15 $$\begin{aligned} v = 0, T= 0, \quad \text {at} \quad \vec {r} = -R. \end{aligned}$$

The radius of the pipe is denoted as $R$. By integrating Eq. (12) for a specified value of $\partial p / \partial z$ and establishing the flow field, the actual pressure gradient can be derived from Eqs. (10), (11), and (12).

### Transform to non-dimensional form

The subsequent dimensional parameters can be utilized to express the governing equations of this model in a dimensionless format.

16 $$\begin{aligned} r^* = \frac{\vec {r}}{R}, v^* = \frac{v}{c}, \theta = \frac{T - T_w}{T_m - T_w}, \end{aligned}$$

In this context, $c$ denotes the reference velocity, while $T_m$ signifies the mean temperature, which may refer to the bulk mean fluid temperature. By incorporating Eq. 16 into the derived equations, one arrives at a series of nonlinear coupled differential equations expressed in the following manner (after the removal of the asterisk*):.

17 $$\begin{aligned} D_1 \left[ \frac{d^2 v}{dr^2} + \frac{1}{r} \frac{dv}{dr} \right] + \Gamma \left[ 3 \left( \frac{dv}{dr} \right) ^2 \frac{d^2 v}{dr^2} + \frac{1}{r} \left( \frac{dv}{dr} \right) ^3 \right] - D_2 H_a^2 v + D_2 E + D_3 \Lambda \theta = C. \end{aligned}$$

18 $$\begin{aligned} D_4 \left[ \frac{d^2 \theta }{dr^2} + \frac{1}{r} \frac{d\theta }{dr} \right] + D_1 \xi _1 \left( \frac{dv}{dr} \right) ^2 + \Gamma \xi _1 \left( \frac{dv}{dr} \right) ^4 + D_2 \xi _1 H_a^2 v^2 - D_2 \xi _2 v + D_2 \xi _3 = 0, \end{aligned}$$

In this context, $\Gamma$ denotes the parameter characterizing a third-grade fluid, while $\xi _1$ represents the Brinkman number. The variable $C$ pertains to the pressure drop, and $H_a$ signifies the Hartmann number. Additionally, $\Lambda$ is identified as the thermal Grashof number, and $\xi _3$ indicates the ratio of joule heating to heat conduction. The parameter $E$ is a dimensionless quantity that reflects the intensity of the electric field, whereas $\xi _2$ represents the heat generation resulting from the interaction between the electrical and magnetic fields and heat conduction. These parameters are defined as follows.

19 $$\begin{aligned} \left. \begin{aligned}&\Gamma = \frac{2\beta _3 c^2}{\mu _f R^2}, \quad C = \frac{\partial p}{\partial z} \frac{R^2}{\mu _f c}, \quad H_a^2 = \frac{\sigma _f}{\mu _f} B_0^2 R^2,\\&\Lambda = \frac{(\rho \beta )_f (T_m - T_w) gR^2}{\mu _f c},\\&\xi _1 = \frac{\mu _f c^2}{\kappa _f (T_m - T_w)}, \quad \xi _2 = \frac{2\sigma _f E B_0 R^2 c}{\kappa _f (T_m - T_w)}, \\&\xi _3 = \frac{\sigma _f R^2 E^2}{\kappa _f (T_m - T_w)},E = \frac{E B_0 \sigma _f R^2}{\mu _f v_0}.\\ \end{aligned} \right\} . \end{aligned}$$

and

20 $$\begin{aligned} D_1 = \frac{\mu _{hnf}}{\mu _f}, \quad D_2 = \frac{\sigma _{hnf}}{\sigma _f}, \quad D_3 = \frac{(\rho \beta )_{hnf}}{(\rho \beta )_f}, \quad D_4 = \frac{\kappa _{hnf}}{\kappa _f}. \end{aligned}$$

The boundary conditions are obtained as:

21 $$\begin{aligned} v(-1) = \theta (-1) = 0, \ \ \text {and} \quad v(1)= \theta (1) = 0. \end{aligned}$$

The thermo-physical properties of density^25^, dynamic viscosity^26^, thermal conductivity^27^, thermal expansion coefficient^28^, and electrical conductivity^29^ are defined below:

(a) Density:

22 $$\begin{aligned} \left. \begin{aligned} \rho _{nf}&= \rho _{f} \left( 1 - \omega _{np1} \right) + \Phi _{1} \rho _{np1}, \\ \rho _{hnf}&= \left( 1 - \omega _{np2} \right) \left( \rho _{f} \left[ 1 - \omega _{np1} \right] + \omega _{np1} \rho _{np1} \right) + \omega _{np2} \rho _{np2}. \end{aligned} \right\} \end{aligned}$$

(b) Dynamic viscosity:

23 $$\begin{aligned} \mu _{nf} = \frac{\mu _f}{(1 - \omega _{np1})^{2.5}}, \quad \mu _{hnf} = \frac{\mu _{nf}}{(1 - \omega _{np2})^{2.5}}. \end{aligned}$$

(c) Thermal conductivity:

24 $$\begin{aligned} \kappa _{nf} = \kappa _{f} \left( \frac{\kappa _{np1} + 2 \kappa _{f} - 2 \omega _{np1} \left( \kappa _{f} - \kappa _{np1} \right) }{\kappa _{np1} + 2 \kappa _{f} + \omega _{np1} \left( \kappa _{f} - \kappa _{np1} \right) } \right) , \end{aligned}$$

25 $$\begin{aligned} \kappa _{hnf} = \kappa _{nf} \left( \frac{\kappa _{np2} + 2 \kappa _{f} - 2 \omega _{np2} \left( \kappa _{f} - \kappa _{np2} \right) }{\kappa _{np2} + 2 \kappa _{f} + \omega _{np2} \left( \kappa _{f} - \kappa _{np2} \right) }) \right\} . \end{aligned}$$

(d) Thermal expansion coefficient:

26 $$\begin{aligned} \left. \begin{aligned}&(\rho \beta )_\textrm{nf} = \Big ( 1-\omega _\textrm{np1}\Big )(\rho \beta )_f + \omega _\textrm{np1}(\rho \beta )_\textrm{np1} \\&(\rho \beta )_\textrm{hnf} = \Big ( 1-\omega _\textrm{np2}\Big )\Big \{\Big ( 1-\omega _\textrm{np1}\Big )(\rho \beta )_f + \omega _\textrm{np1}(\rho \beta )_\textrm{np1}\Big \} + \omega _\textrm{np2}(\rho \beta )_\textrm{np2}. \end{aligned} \right\} \end{aligned}$$

(e) Electric conductivity:

27 $$\begin{aligned} \left. \begin{aligned} \sigma _{nf}&= \sigma _{f} \left( \frac{\sigma _{np1} (1 + 2\omega _{np1}) + 2\sigma _{f} (1 - \omega _{np1})}{\sigma _{np1} (1 - \omega _{np1}) + \sigma _{f} (2 + \omega _{np1})} \right) , \\ \sigma _{hnf}&= \sigma _{nf} \left( \frac{\sigma _{np2} (1 + 2\omega _{np2}) + 2\sigma _{f} (1 - \omega _{np2})}{\sigma _{np2} (1 - \omega _{np2}) + \sigma _{f} (2 + \omega _{np2})} \right) . \end{aligned} \right\} \end{aligned}$$

**Table 1:** The thermo-physical properties of sodium alginate (*SA*), zinc oxide (*ZnO*), and copper oxide (*CuO*) nanoparticles^30,31^.

Material	$\rho$ (kg m$^{-3}$)	*c* (J kg$^{-1}$ K$^{-1}$)	$\beta$ (K$^{-1}$)	$\kappa$ (W m$^{-1}$ K$^{-1}$)	$\sigma$ (S m$^{-1}$)
*SA*	989	4175	$99 \times 10^{-5}$	0.6376	$2.64 \times 10^{-4}$
*ZnO*	5600	495.2	$6.15 \times 10^{-6}$	13	$5.4 \times 10^{-2}$
*CuO*	6500	540	$29 \times 10^{-5}$	18	$2.7 \times 10^{-8}$

In this context, $(\omega _\textrm{np1}, \omega _\textrm{np2})$ denotes the volume fractions of zinc oxide (ZnO) and copper oxide (CuO) nanoparticles, with the subscripts nf representing the nanofluid. Specifically, $(\omega _\textrm{np1}, \omega _\textrm{np2})$ corresponds to the first nanoparticle (zinc oxide) and the second nanoparticle (copper oxide). The aforementioned model has been employed to evaluate the computational outcomes for both hybrid nanofluids and nanofluids. The thermo-physical properties of sodium alginate (*SA*), zinc oxide (*ZnO*), and copper oxide (*CuO*) nanoparticles is given in Table 1. This modeling approach has been utilized by Bhatti and Abdelsalam^29^, Jakeer et al.^30^, and Armaghani et al.^31^ to investigate the dynamics of hybrid nanofluid flows. The skin friction coefficient and the Nusselt number are as given below,

28 $$\begin{aligned} \begin{aligned} S&= \left[ \mu _1 \frac{dv}{d\vec {r}} + \mu _3 \left( \frac{dv}{d\vec {r}}\right) ^3 \right] _{r=R}, \\ N&= - k \left. \frac{d T}{d\vec {r}} \right| _{r=R}. \end{aligned} \end{aligned}$$

The important physical quantities relevant to hybrid nanofluids, including the skin friction coefficient and the Nusselt number, are expressed in a dimensionless format as follows:

29 $$\begin{aligned} \begin{aligned} S&= \left[ D_{1}\frac{\textrm{d}v}{\textrm{d}r} + \Gamma \left( \frac{\textrm{d}v}{\textrm{d}r}\right) ^{3}\right] \Bigg |_{r=1},\\ N&= -D_{4}\left. \frac{\textrm{d}\theta }{\textrm{d}r}\right| _{r=1}. \end{aligned} \end{aligned}$$

## Physics-driven methodology

Deep Neural Networks (DNNs) are a powerful technique that folds a system’s governing laws directly into the learning framework. This particular approach uses the assumption of physical laws to guide the learning process by embedding differential equations in the form of constraints that are met during the training of unsupervised deep neural networks. DNNs can be of particular benefit in solving problems in scientific computing, engineering, and physics were classical numerical methods are incapable or impractical. Here, we describe the methodology and the underlying ideas of the DNNs for solving differential equations. A partial differential equation (PDE) problem is generally stated as follows:

30 $$\begin{aligned} \mathcal {L}(u)(x)= & f(x), \quad x \in \Omega \end{aligned}$$

31 $$\begin{aligned} \mathcal {B}(u)(x)= & g(x), \quad x \in \partial \Omega \end{aligned}$$

In this context, $\mathcal {L}$ a differential operator is used, $u(x)$ the unknown solution is represented, $f(x)$ a source term is present, $\mathcal {B}$ boundary conditions are defined, $\Omega$ the domain and its $\partial \Omega$ boundary are specified. The deep neural network leverages a neural network $u_\theta (x)$ representation to approximate the solution $u(x)$, with $\theta$ adjustable parameters. The training process of the network ensures adherence to both the boundary conditions and the differential equation.

### Loss function construction

The loss function of the deep neural network (DNN) incorporates the differential equation and boundary conditions. Typically, it consists of two main components:

PDE Loss: This component assesses the extent to which the neural network satisfies the conditions set by the differential equation.

32 $$\begin{aligned} \mathcal {L}{oss}_{\text {PDE}} = \frac{1}{N_f} \sum _{i=1}^{N_f} \left| \mathcal {L}(u_\theta )(x_i) - f(x_i) \right| ^2 \end{aligned}$$

Here, the points $x_i$ are utilized as collocation points sourced from the domain $\Omega$.

Boundary Loss: Confirms that the solution satisfies the required boundary conditions.

33 $$\begin{aligned} \mathcal {L}{oss}_{\text {BC}} = \frac{1}{N_b} \sum _{i=1}^{N_b} \left| \mathcal {B}(u_\theta )(x_i) - g(x_i) \right| ^2 \end{aligned}$$

Here, the term $x_i$ indicates the points derived from the boundary $\partial \Omega$. The complete loss is represented by the weighted aggregation of these elements.

34 $$\begin{aligned} \mathcal {L}{oss} = \lambda _{\text {PDE}} \cdot \mathcal {L}{oss}_{\text {PDE}} + \lambda _{\text {BC}} \cdot \mathcal {L}{oss}_{\text {BC}} \end{aligned}$$

where $\lambda _{\text {PDE}}$ and $\lambda _{\text {BC}}$ represents weighting factors. The prediction of the velocity and temperature profiles in the EMHD problem is achieved through the use of an unsupervised deep neural network. The governing equations, along with their boundary conditions, are converted into a mean squared error format, which is then utilized as the loss function. This loss function aggregates the losses obtained from the differential equations and the boundary conditions. The L-BFGS algorithm is applied to train and minimize this loss function, resulting in the identification of the most effective weights and biases after the network has undergone training. The implementation of this unsupervised deep neural network technique on the designated system results in,

35 $$\begin{aligned} \mathcal {L}=\mathcal {L}_{PDE}+\mathcal {L}_{BC}, \end{aligned}$$

where loss function for differential equations are the following,

36 $$\begin{aligned} \mathcal {L}_{Eq1}=\frac{1}{N} \sum _{i=1}^N\left| D_1 \left[ \frac{d^2\hat{v_i}}{dr^2} + \frac{1}{r}\frac{dv}{dr} \right] + \Gamma \left[ 3 \left( \frac{d\hat{v_i}}{dr} \right) ^2 \frac{d^2\hat{v_i}}{dr^2} + \frac{1}{r} \left( \frac{d\hat{v_i}}{dr} \right) ^3 \right] - D_2 H_a^2 \hat{v_i} + D_2 E + D_3 \Lambda \theta -C\right| ^2, \end{aligned}$$

37 $$\begin{aligned} \mathcal {L}_{Eq2}=\frac{1}{N} \sum _{i=1}^N\left| D_4 \left[ \frac{d^2 {\hat{\theta }}_i}{dr^2} + \frac{1}{r} \frac{d{\hat{\theta }}_i}{dr} \right] + D_1 \xi _1 \left( \frac{d\hat{v_i}}{dr} \right) ^2 + \Gamma \xi _1 \left( \frac{d\hat{v_i}}{dr} \right) ^4 + D_2 \xi _1 H_a^2 \hat{v_i}^2 - D_2 \xi _2 \hat{v_i} + D_2 \xi _3\right| ^2, \end{aligned}$$

and the loss function for the boundary conditions are given by,

38 $$\begin{aligned} \mathcal {L}_{BCs}=\left| \hat{v}(1)\right| ^2+\left| \hat{\theta }(1)\right| ^2+\left| { \frac{d\hat{v}}{dr}}(0)\right| ^2+\left| {\frac{d{\hat{\theta }}}{dr}}(0)\right| ^2. \end{aligned}$$

The solution for temperature and velocity profile is then obtained by evaluating the deep neural network function in summation form with the best set of weights and biases in the last epoch.

### Training the neural network

**Fig. 2:**
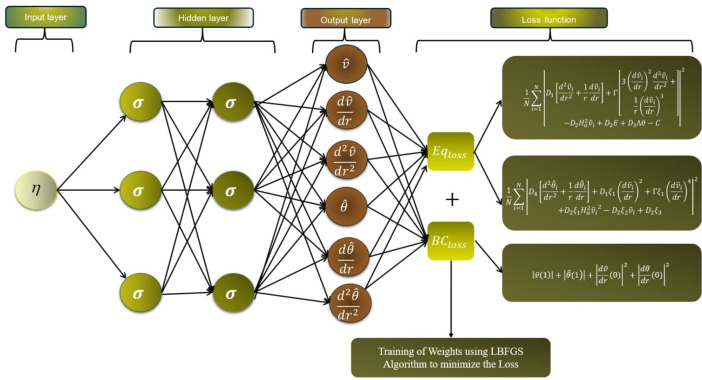
Architecture of the deep neural network for the given problem.

We employed the L-BFGS gradient-based optimization algorithm to train the neural network, aiming to identify the optimal set of weights for solution prediction. Automatic differentiation facilitates the computation of gradients of the loss function concerning the network parameters $\theta$. The network undergoes iterative updates to reduce the loss function. Figure 2 shows the whole procedure to predict the solution for the EMHD flow problem. While algorithm 1 shows the pseudo framework to execute the whole prcoess for prediction of the EMHD flow problem.

**Algorithm 1:**
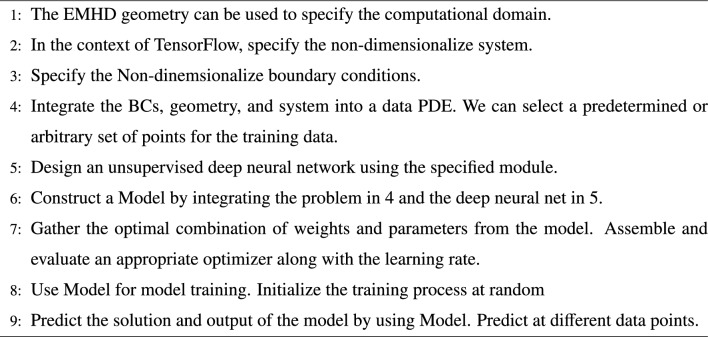
EMHD Nanofluid Framework Utilizing Unsupervised Deep Neural Networks.

**Table 2:** Train Loss and Test Loss for the parameter *Ha* for four different cases using the designed deep neural network.

Cases	Eq1	Eq2	BC1	BC2	BC3	BC4	Mean
Train Loss							
C-1	1.10e-06	1.30e-06	2.05e-12	3.20e-08	1.72e-12	3.89e-09	2.44e-06
C-2	2.45e-06	5.01e-07	8.88e-14	2.67e-08	4.30e-13	1.83e-08	3.00e-06
C-3	1.33e-06	9.81e-06	7.06e-11	7.47e-08	3.99e-12	3.06e-07	1.15e-05
C-4	1.02e-08	1.14e-07	3.55e-15	1.77e-11	3.92e-13	5.50e-10	1.25e-07
Test Loss							
C-1	1.16e-06	1.11e-06	2.05e-12	3.20e-08	1.72e-12	3.89e-09	2.30e-06
C-2	2.35e-06	5.06e-07	8.88e-14	2.67e-08	4.30e-13	1.83e-08	2.90e-06
C-3	1.36e-06	9.52e-06	7.06e-11	7.47e-08	3.99e-12	3.06e-07	1.13e-05
C-4	8.51e-09	1.06e-07	3.55e-15	1.77e-11	3.92e-13	5.50e-10	1.15e-07

## Predicted results and discussion

**Fig. 3:**
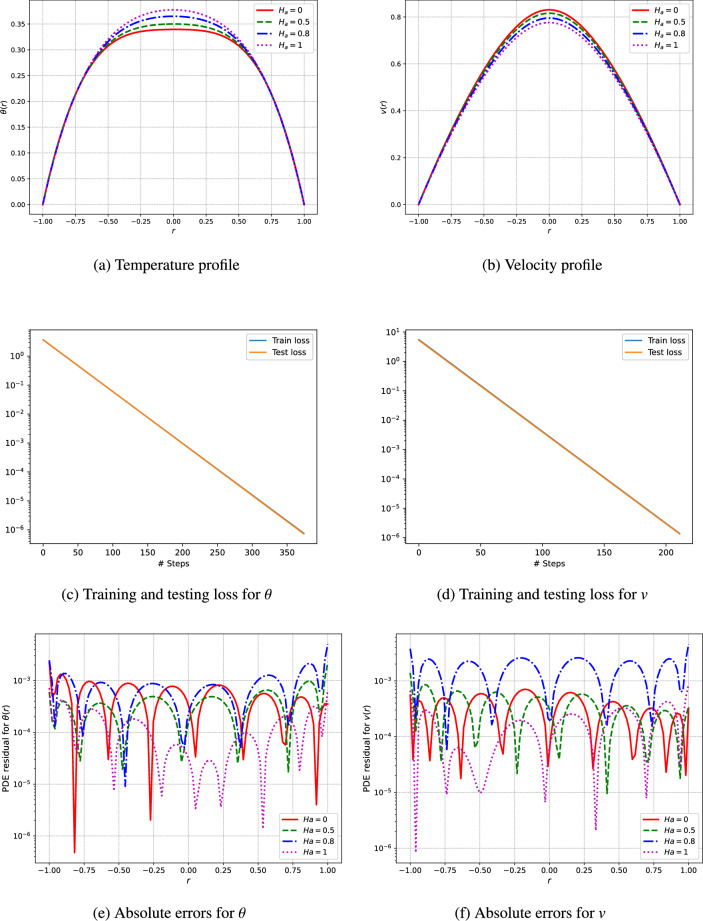
The velocity and temperature profile of the fluid and training and testing loss using the unsupervised deep neural network.

**Fig. 4:**
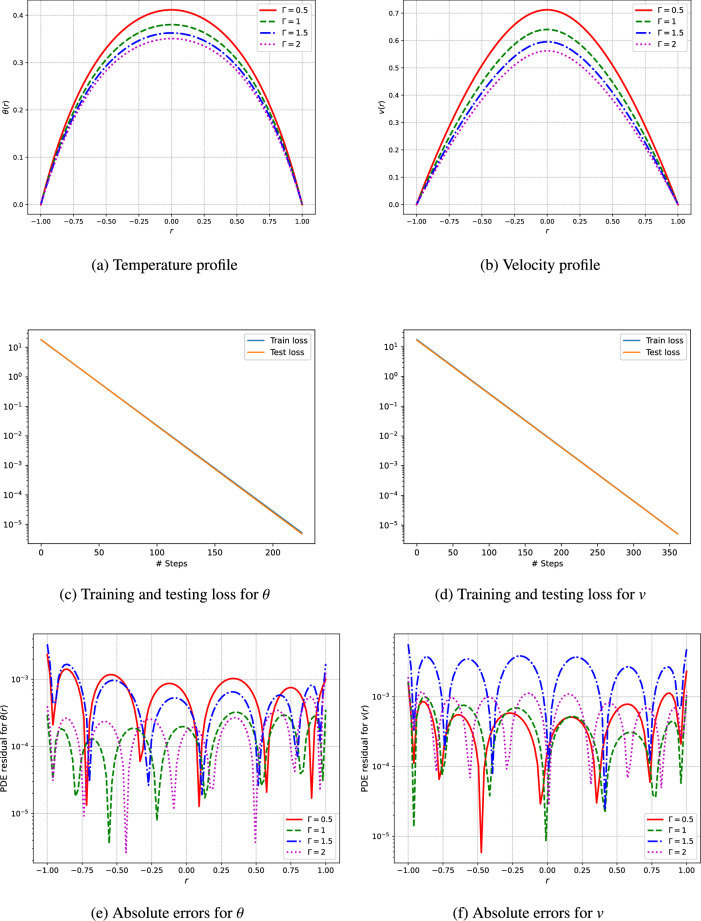
The velocity and temperature profile of the fluid and training and testing loss using the unsupervised deep neural network.

**Fig. 5:**
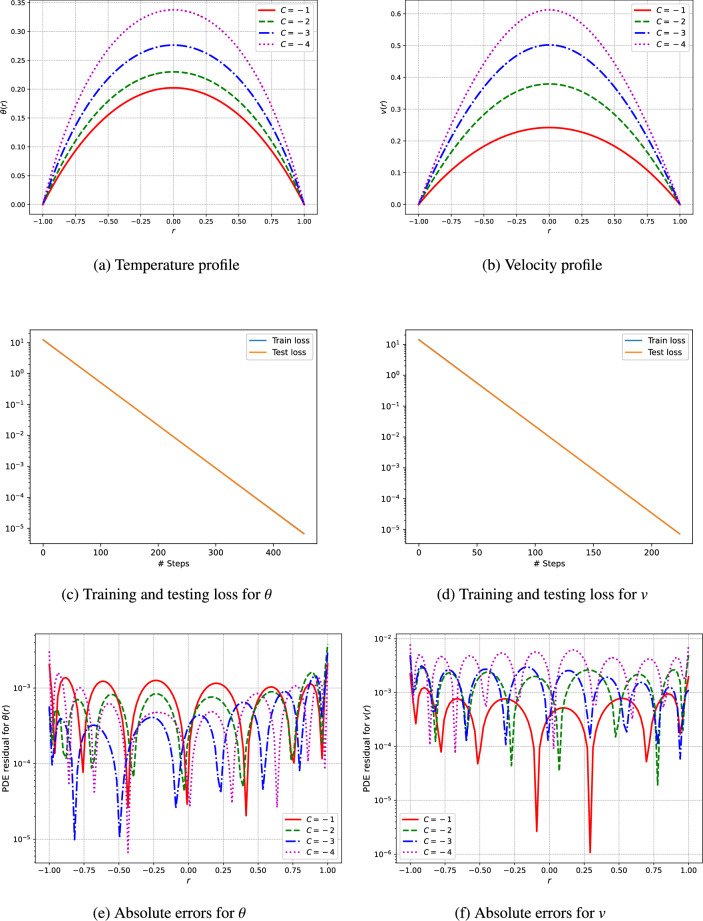
The velocity and temperature profile of the fluid and training and testing loss using the unsupervised deep neural network.

**Fig. 6:**
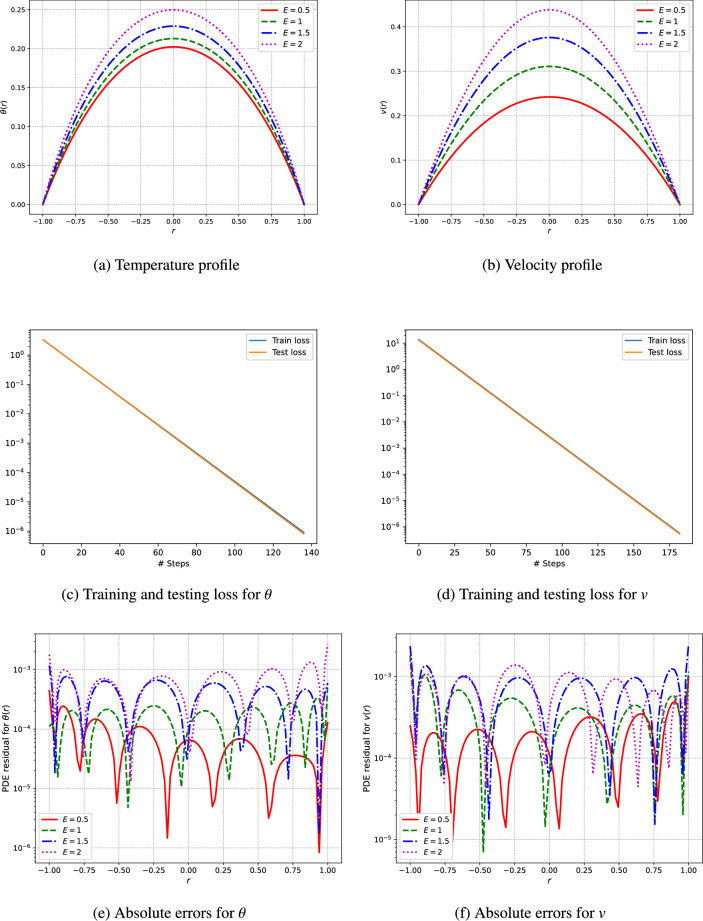
The velocity and temperature profile of the fluid and training and testing loss using the unsupervised deep neural network.

**Fig. 7:**
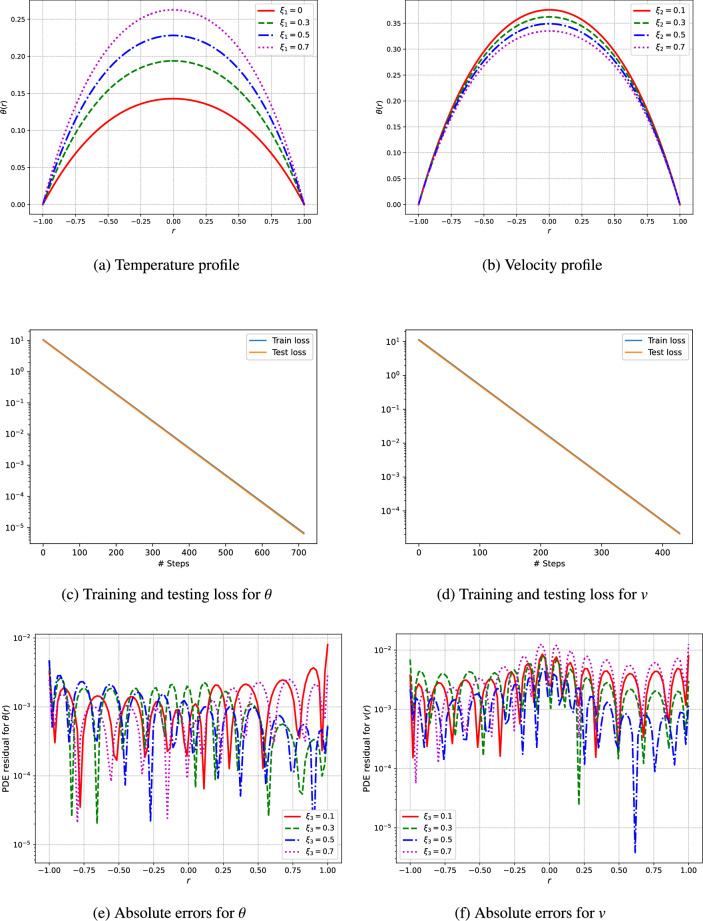
The velocity and temperature profile of the fluid and training and testing loss using the unsupervised deep neural network.

An unsupervised deep neural network is utilized to predict the profiles of temperature and velocity. The simulations are conducted using Python and the TensorFlow library for automatic differentiation, while the L-BFGS algorithm is employed for the training of the network. The model architecture features a single input layer, three fully connected hidden layers containing 50 neurons each, and a single output layer. To analyze the visual results, the outcomes from the deep neural networks are computed through the network’s training procedure. This research addresses various parameters, including the Hartmann number $(H_a)$, the third-grade fluid parameter $(\Gamma )$, the Brinkman number $(\xi _1)$, the heat generation parameters related to electric and magnetic fields $(\xi _2)$, the electric field parameter (*E*), and the pressure (*C*) as shown in Figs. 3, 4, 5, 6, 7. We have considered the parameters values as ($H_a = 1.5$, $\Gamma = 0.5$, $C = -1$, $E = 0.5$, $\xi _1 = \xi _2 = \xi _3 = 1$).

The velocity profiles depicted in Fig. 3b demonstrate the impact of the Hartmann number $(H_a)$ on the behavior of fluid flow. As $H_a$ increases, the velocity gradient near the boundaries becomes more pronounced, indicating a stronger magnetic field influence on the fluid’s motion. This aligns with the known characteristics of magnetohydrodynamic flows, where higher $H_a$ facilitate the formation of boundary layers and decrease the velocity of the core flow. Furthermore, the temperature profiles in Fig. 3a provide insights into the thermal distribution within the fluid at different Hartmann numbers. A higher $H_a$ results in a more uniform temperature distribution, suggesting that the magnetic field enhances thermal diffusion.

The results of our investigation are presented in Fig. 4, showcasing the velocity and temperature profiles of a third-grade fluid, as well as the training and testing loss obtained from an unsupervised deep neural network. The parameter $\Gamma$, which characterizes the non-Newtonian properties of the fluid, was adjusted to evaluate its impact on the fluid dynamics and thermal performance.

The velocity profiles showcase the influence of $\Gamma$ on fluid dynamics. As $\Gamma$ rises, the fluid exhibits more pronounced non-Newtonian characteristics, leading to changes in the velocity distribution. Specifically, larger $\Gamma$ values produce a more uniform velocity profile across the fluid, indicating amplified shear-thinning properties. This behavior is typical of third-grade fluids, where viscosity decreases with increasing shear rate. Our research findings are depicted in Fig. 4, which showcases the velocity and temperature profiles of a third-grade fluid, as well as the training and testing loss from an unsupervised deep neural network. The parameter $\Gamma$, which characterizes the non-Newtonian properties of the fluid, was adjusted to evaluate its impact on fluid dynamics and thermal performance.

The temperature profiles (Fig. 4a) illustrate the thermal distribution within the fluid at varying $\Gamma$ values. An increase in $\Gamma$ results in a more uniform temperature distribution, indicating improved thermal conductivity and heat transfer efficiency. This observation aligns with the enhanced mixing and reduced viscosity associated with higher $\Gamma$ values, which promote better heat dissipation. The fluctuation of Gamma has important ramifications for practical applications, especially in the development of industrial processes that involve non-Newtonian fluids. Increased values of $\Gamma$ can lead to improvements in heat transfer and flow consistency, yet they also demand careful evaluation of the mechanical energy input as a result of modified viscosity characteristics. Thus, optimizing $\Gamma$ is vital for realizing the intended flow and thermal attributes in third-grade fluid systems.

Figure 5 presents the outcomes of our study, illustrating the profiles of velocity, temperature, and pressure drop of the fluid, the absolute errors, along with the training and testing loss achieved through an unsupervised deep neural network. These profiles enhance our understanding of the fluid dynamics and thermal behavior across various conditions. The velocity profiles illustrated in Fig. 5b determines the characteristics of fluid flow under varying conditions. These profiles highlight the alterations in velocity distribution throughout the fluid domain. It is noted that elevated velocities are present near the center of the flow, gradually diminishing towards the boundaries, a pattern commonly associated with laminar flow conditions.The form of the velocity profile is shaped by factors including fluid viscosity and the specific boundary conditions. The thermal distribution within the fluid is depicted in the temperature profiles shown in Fig. 5a. These profiles illustrate how temperature varies throughout the fluid, with increased temperatures usually detected near heat sources or in areas with significant energy dissipation. The uniformity of this temperature distribution is determined by the fluid’s thermal conductivity and its flow properties.

The results of our analysis are illustrated in Fig. 6, illustrating the velocity and temperature profiles of the fluid, residual errors, as well as the training and testing loss achieved through an unsupervised deep neural network. The parameter E, a dimensionless value indicative of the electric field’s intensity, was modified to assess its effect on the fluid dynamics and thermal behavior. Figure 6b depicts the velocity profile, which highlights the influence of electric field intensity on fluid flow behavior. As the E-field strength increases, the velocity distribution throughout the fluid experiences a notable change. Elevated E-field values correlate with an increase in flow velocity, attributable to the additional force exerted by the electric field on the charged particles within the fluid. This phenomenon is especially pronounced in electrohydrodynamic flows, where electric fields can trigger movement in fluids that would otherwise remain stationary. Figure 6a depicts the temperature profiles that illustrate the thermal distribution within the fluid under varying electric field intensities. An increase in the electric field intensity yields a more uniform temperature distribution, implying enhanced thermal mixing and improved heat transfer efficiency. This observation aligns with the enhanced fluid motion generated by the electric field, which promotes better heat dissipation and reduces temperature gradients. Variations in electric field intensity have significant implications for practical applications, especially in the design of EHD pumps, cooling systems, and microfluidic devices. Higher values of electric field can promote improved fluid dynamics and thermal transfer, but they also necessitate careful evaluation of energy efficiency and the potential for electrical failure. Consequently, optimizing the electric field intensity is crucial for achieving optimal performance in EHD systems.

Figure 7 showcases the results of our investigation, depicting the velocity and temperature bahviour of the fluid, absolute errors, as well as the training and testing loss achieved through an unsupervised deep neural network for the parameters $\xi _1$ and $\xi _2$. The temperature distribution within the fluid, depicted in Fig. 7a and b, is affected by varying values of $\xi _1$ and $\xi _2$. The heat generation parameter, $\xi _2$, is crucial in determining the temperature profile. Increasing $\xi _2$ leads to greater internal heat generation, resulting in higher temperatures throughout the fluid. This phenomenon is particularly pronounced in regions where the interaction between electrical and magnetic fields is significant, thereby enhancing heat conduction and resulting in more uniform temperature profiles.

**Discussion on the Performance of DNN**

The training and testing loss and absolute errors curves depicted in Figs. 3, 4, 5, 6, 7 and Tables 2, 3, 4, 5, 6, 7 demonstrate the effectiveness of the unsupervised deep neural network in modeling the nonlinear dynamics of fluids and thermal properties under diverse conditions. Specifically, the results for the Hartmann number, presented in Fig. 3c and d alongside Table 2 and training testing losses ranges form $1.15e^{-05}$ to $3.55e^{-15}$, indicate that the convergence of the loss curves reflects the model’s skill in predicting velocity and temperature profiles influenced by magnetic fields. Similarly, for the parameter denoted as $\Gamma$ in Fig. 4c and d and Table 3, where training and testing losses ranges from $9.76e^{-06}$ to $3.55e^{-13}$ the low testing loss values signify the model’s ability to accurately forecast the behavior of non-Newtonian fluids. Moreover, regarding pressure drops represented in Fig. 5c and d and Table 4, moreover the training and testing losses recorded in the range $6.8e^{-06}$ to $3.55e^{-13}$, the model effectively captures energy losses, which is evident from the minimal testing loss, underscoring its robustness.In reference to the electric field intensity parameter *E* depicted in Fig. 6c and d and Table 5, the strong convergence of the loss curves suggests that the model yields precise predictions of electrohydrodynamic phenomena, with training and testing losses ranging from $1.09e^{-06}$ to $1.42e^{-14}$. Lastly, concerning the Brinkman number ($\xi _1$) and heat generation ($\xi _2$) in Fig. 7c and d and Tables 6 and 7, the model successfully predicts the interplay between viscous dissipation and internal heat generation, as indicated by the low values of testing loss which are in the range $2.18e^{-05}$ to $1.04e^{-11}$. Collectively, the consistent performance across all parameters accentuates the model’s generalization capability and its potential for delivering precise predictions in complex scenarios.

**Table 3:** Train Loss and Test Loss for the parameter $\Gamma$ for four different cases using the designed deep neural network.

Cases	Eq1	Eq2	BC1	BC2	BC3	BC4	Mean
Train Loss							
C-1	7.31e-06	1.31e-06	1.64e-10	1.17e-07	1.77e-11	4.21e-09	8.74e-06
C-2	4.77e-06	4.89e-06	1.24e-10	5.53e-08	6.88e-12	4.88e-08	9.76e-06
C-3	6.78e-06	6.26e-07	8.88e-12	1.38e-07	3.55e-13	1.05e-09	7.55e-06
C-4	3.01e-06	2.20e-06	1.34e-10	5.00e-09	2.05e-11	4.55e-09	5.23e-06
Test Loss							
C-1	6.98e-06	1.34e-06	1.64e-10	1.17e-07	1.77e-11	4.21e-09	8.45e-06
C-2	4.56e-06	4.43e-06	1.24e-10	5.53e-08	6.88e-12	4.88e-08	9.10e-06
C-3	6.36e-06	5.41e-07	8.88e-12	1.38e-07	3.55e-13	1.05e-09	7.04e-06
C-4	2.72e-06	2.03e-06	1.34e-10	5.00e-09	2.05e-11	4.55e-09	4.76e-06

**Table 4:** Train Loss and Test Loss for the parameter *C* for four different cases using the designed deep neural network.

Cases	Eq1	Eq2	BC1	BC2	BC3	BC4	Mean
Train Loss							
C-1	2.51e-07	3.82e-06	9.67e-11	6.05e-09	3.55e-13	5.90e-08	4.14e-06
C-2	3.10e-06	1.11e-06	1.23e-10	7.85e-08	1.15e-12	3.27e-08	4.32e-06
C-3	4.42e-06	1.11e-06	1.15e-11	1.60e-07	2.79e-12	4.94e-08	5.74e-06
C-4	6.11e-06	4.94e-07	4.07e-11	1.95e-07	2.22e-12	5.40e-09	6.80e-06
Test Loss							
C-1	2.35e-07	3.64e-06	9.67e-11	6.05e-09	3.55e-13	5.90e-08	3.94e-06
C-2	3.05e-06	1.05e-06	1.23e-10	7.85e-08	1.15e-12	3.27e-08	4.21e-06
C-3	4.46e-06	1.07e-06	1.15e-11	1.60e-07	2.79e-12	4.94e-08	5.74e-06
C-4	6.02e-06	4.21e-07	4.07e-11	1.95e-07	2.22e-12	5.40e-09	6.64e-06

**Table 5:** Train Loss and Test Loss for the parameter *E* for four different cases using the designed deep neural network.

Cases	Eq1	Eq2	BC1	BC2	BC3	BC4	Mean
Train Loss							
C-1	2.61e-08	1.06e-06	2.11e-11	1.25e-10	2.22e-11	1.20e-08	1.09e-06
C-2	9.98e-08	8.51e-07	4.11e-12	5.96e-10	3.87e-12	2.24e-08	9.74e-07
C-3	3.33e-07	4.52e-07	6.27e-12	1.49e-08	1.28e-13	4.83e-09	8.04e-07
C-4	6.87e-08	8.25e-07	1.07e-11	2.81e-09	1.42e-14	8.16e-09	9.04e-07
Test Loss							
C-1	2.32e-08	9.83e-07	2.11e-11	1.25e-10	2.22e-11	1.20e-08	1.02e-06
C-2	9.77e-08	8.20e-07	4.11e-12	5.96e-10	3.87e-12	2.24e-08	9.41e-07
C-3	3.33e-07	4.17e-07	6.27e-12	1.49e-08	1.28e-13	4.83e-09	7.70e-07
C-4	6.85e-08	7.29e-07	1.07e-11	2.81e-09	1.42e-14	8.16e-09	8.08e-07

**Table 6:** Train Loss and Test Loss for the parameter $\xi _1$ for four different cases using the designed deep neural network.

Cases	Eq1	Eq2	BC1	BC2	BC3	BC4	Mean
Train Loss							
C-1	2.42e-05	3.91e-06	1.45e-10	2.33e-09	8.13e-10	1.22e-09	2.81e-05
C-2	1.54e-05	8.27e-06	2.91e-08	2.25e-08	7.19e-14	2.66e-11	2.37e-05
C-3	4.42e-05	3.51e-06	4.27e-08	1.93e-08	2.99e-10	9.68e-10	4.78e-05
C-4	2.43e-05	5.39e-06	4.81e-09	4.97e-10	8.46e-10	1.36e-09	2.97e-05
Test Loss							
C-1	2.29e-05	3.33e-06	1.45e-10	2.33e-09	8.13e-10	1.22e-09	2.63e-05
C-2	1.50e-05	6.43e-06	2.91e-08	2.25e-08	7.19e-14	2.66e-11	2.15e-05
C-3	4.20e-05	3.60e-06	4.27e-08	1.93e-08	2.99e-10	9.68e-10	4.57e-05
C-4	2.34e-05	4.07e-06	4.81e-09	4.97e-10	8.46e-10	1.36e-09	2.75e-05

**Table 7:** Train Loss and Test Loss for the parameter $\xi _2$ for four different cases using the designed deep neural network.

Cases	Eq1	Eq2	BC1	BC2	BC3	BC4	Mean
Train Loss							
C-1	7.28e-06	5.26e-06	6.36e-09	2.41e-09	9.53e-10	5.24e-10	1.26e-05
C-2	5.74e-06	2.63e-07	1.04e-11	8.43e-11	6.27e-10	1.57e-10	6.00e-06
C-3	6.35e-06	4.06e-06	3.99e-08	3.70e-08	1.08e-10	1.20e-10	1.05e-05
C-4	1.73e-05	4.52e-06	3.64e-09	2.93e-09	1.85e-10	1.91e-10	2.18e-05
Test Loss							
C-1	7.16e-06	4.13e-06	6.36e-09	2.41e-09	9.53e-10	5.24e-10	1.13e-05
C-2	5.47e-06	2.27e-07	1.04e-11	8.43e-11	6.27e-10	1.57e-10	5.70e-06
C-3	6.32e-06	3.40e-06	3.99e-08	3.70e-08	1.08e-10	1.20e-10	9.79e-06
C-4	1.66e-05	4.24e-06	3.64e-09	2.93e-09	1.85e-10	1.91e-10	2.09e-05

## Conclusion

This study investigates the potential advantages of hybrid nanofluids, specifically third-grade sodium alginate fluid, in geothermal energy extraction. Zinc oxide (ZnO) and copper oxide (CuO) nanoparticles are incorporated to regulate the flow dynamics. The sodium alginate fluid exhibits electrical conductivity due to the presence of transverse magnetic and electric fields. An incompressible, electrically conducting fluid flows through a pipe, with additional considerations for convection, viscous dissipation, and Joule heating. To solve the governing nonlinear differential equations, a deep neural network (DNN) approach is employed, providing highly accurate predictions for the temperature and velocity of EMHD flow. The key findings of this study are summarized as follows:

- The non-Newtonian nature of the fluid and the application of a magnetic field result in a decreasing trend in the velocity profiles.
- The electric field and the thermal Grashof number significantly enhance the velocity profile, creating a symmetrical pattern.
- The inclusion of nanoparticles increases the fluid’s viscosity, which tends to reduce its flow velocity.
- The thermal profile increases notably with higher magnetic field strength, third-grade fluid parameter, and Brinkman number.
- The addition of nanoparticles reduces the thermal profile across the entire pipe length, while the absence of copper oxide nanoparticles leads to the highest thermal profile.
- Both the velocity and thermal characteristics are similarly influenced by the pressure drop within the system.
- One of the application this model is of geothermal heat exchangers, where enhanced thermal conductivity and flow control can significantly improve energy extraction efficiency.
- The accuracy of the proposed deep neural network methodology is in the range of $10^{-03}$ to $10^{-13}$ for different cases.

The study highlights the importance of advanced computational techniques, such as deep neural networks (DNNs), in solving highly nonlinear systems and advancing research in fluid dynamics and thermal energy applications.

### Future Directions

In the future, we will apply the given methodology to 3D complex problems and work to improve the accuracy and convergence of the DNN method for these challenges. Additionally, the method will be tested on more real-world engineering problems.

## Data Availability

No datasets were generated or analyzed during the current study.
